# Adverse Events Due to Insomnia Drugs Reported in a Regulatory Database and Online Patient Reviews: Comparative Study

**DOI:** 10.2196/13371

**Published:** 2019-11-08

**Authors:** Jill S Borchert, Bo Wang, Muzaina Ramzanali, Amy B Stein, Latha M Malaiyandi, Kirk E Dineley

**Affiliations:** 1 Chicago College of Pharmacy Midwestern University Downers Grove, IL United States; 2 Chicago College of Osteopathic Medicine Midwestern University Downers Grove, IL United States; 3 Office of Research and Sponsored Programs Midwestern University Glendale, AZ United States; 4 College of Graduate Studies Midwestern University Downers Grove, IL United States

**Keywords:** drug safety, drug ineffective, postmarketing, pharmacovigilance, internet, pharmacoepidemiology, adverse effect, hypnotic, insomnia, patient-reported outcomes

## Abstract

**Background:**

Patient online drug reviews are a resource for other patients seeking information about the practical benefits and drawbacks of drug therapies. Patient reviews may also serve as a source of postmarketing safety data that are more user-friendly than regulatory databases. However, the reliability of online reviews has been questioned, because they do not undergo professional review and lack means of verification.

**Objective:**

We evaluated online reviews of hypnotic medications, because they are commonly used and their therapeutic efficacy is particularly amenable to patient self-evaluation. Our primary objective was to compare the types and frequencies of adverse events reported to the Food and Drug Administration Adverse Event Reporting System (FAERS) with analogous information in patient reviews on the consumer health website Drugs.com. The secondary objectives were to describe patient reports of efficacy and adverse events and assess the influence of medication cost, effectiveness, and adverse events on user ratings of hypnotic medications.

**Methods:**

Patient ratings and narratives were retrieved from 1407 reviews on Drugs.com between February 2007 and March 2018 for eszopiclone, ramelteon, suvorexant, zaleplon, and zolpidem. Reviews were coded to preferred terms in the Medical Dictionary for Regulatory Activities. These reviews were compared to 5916 cases in the FAERS database from January 2015 to September 2017.

**Results:**

Similar adverse events were reported to both Drugs.com and FAERS. Both resources identified a lack of efficacy as a common complaint for all five drugs. Both resources revealed that amnesia commonly occurs with eszopiclone, zaleplon, and zolpidem, while nightmares commonly occur with suvorexant. Compared to FAERS, online reviews of zolpidem reported a much higher frequency of amnesia and partial sleep activities. User ratings were highest for zolpidem and lowest for suvorexant. Statistical analyses showed that patient ratings are influenced by considerations of efficacy and adverse events, while drug cost is unimportant.

**Conclusions:**

For hypnotic medications, online patient reviews and FAERS emphasized similar adverse events. Online reviewers rated drugs based on perception of efficacy and adverse events. We conclude that online patient reviews of hypnotics are a valid source that can supplement traditional adverse event reporting systems.

## Introduction

Postmarketing surveillance is coordinated by regulatory bodies that use passive collection systems to monitor the occurrence of drug toxicities. The Food and Drug Administration (FDA) Adverse Event Reporting System (FAERS) relies on drug manufacturers, health care professionals, and the patients themselves to report instances of adverse events in the form of an Individual Case Safety Report [1,2]. Although manufacturers are obligated to report adverse events, reporting by health care professionals and patients is voluntary. Consequently, a large amount of safety data never reach regulators [3-5], which underscores the need for additional mechanisms of pharmacovigilance.

Over the past decade, a rapidly expanding body of research has focused on the abundance of health data generated by patient online activities [6-10]. These include data collected from Web browser search habits [11,12], commentary that appears on social media such as Facebook and Twitter [13-16], and information revealed on internet forums [17,18]. The field of inquiry concerned with mining these resources for the purposes of improving public health has been referred to as “infodemiology” [19].

One type of Web datum enriched with drug safety information appears on websites such as WebMD, AskaPatient, and Drugs.com in the form of patient drug reviews. In a few sentences or a short paragraph, the patient shares his/her personal experience with efficacy, adverse events, and other issues related to the use of a given drug. Researchers have begun to explore how these reviews might improve pharmacovigilance. For example, online reviews have been used to show that emotional and behavioral effects are prominent considerations for users of antidepressants and antipsychotics [20,21]. Others found that online reviewers tend to describe less serious adverse events than those described by FAERS reports [22]. Web reviews can also help assess illicit drugs that fall outside of conventional trial evaluation [23], although evidence suggests that online commentary tends to minimize the dangers of habit-forming drugs [24]. Taken together, these studies suggest that online patient drug reviews can enhance traditional pharmacovigilance mechanisms by offering quick collection of information from large, diverse populations, followed by rapid dissemination in a form that is accessible to the lay community.

This study compares information contributed by patients to an online drug information website with adverse event data collected by the FDA. We focused on a particular set of FDA-approved hypnotics because they are common drugs used almost exclusively for the treatment of sleep disorders [25,26]. This minimized concerns over variable patient experience that arises when the same drugs are used for different therapeutic applications. In addition, hypnotics are well suited to patient evaluation, because the goal of therapy is simple, and most unwanted effects are easily identified. In our primary objective, we compared the frequencies of adverse events reported in FAERS with analogous information that appeared in patient reviews on the website Drugs.com. Secondary objectives were to describe patient reports of efficacy and adverse events and determine whether cost, effectiveness, or adverse events influenced user ratings of hypnotic medications.

## Methods

### Hypnotics

Five hypnotics were selected for this study. Eszopiclone, zaleplon, and zolpidem are a class of benzodiazepine receptor agonist known as Z-drugs and are the most commonly prescribed class of hypnotics [27]. Ramelteon is a melatonin receptor agonist that promotes sleep via activation of the melatonin 1 receptor subtype (MT1) [28]. Suvorexant promotes sleep by blocking OX1 and OX2 orexin receptors and is the first dual orexin receptor antagonist (DORA) approved for clinical use [29]. Other drugs used for insomnia (eg, benzodiazepines, trazodone, antihistamines, and melatonin) were excluded because those agents are frequently used for indications unrelated to sleep disorder or they are not FDA approved for the treatment of sleep disorders.

### Online Reviews

A total of 1407 publicly available online drug reviews concerning either eszopiclone (n=239), ramelteon (n=72), suvorexant (n=324), zaleplon (n=82), or zolpidem (n=690), dated from February 2007 to March 2018, were retrieved from the website Drugs.com, a drug information platform for consumers and health care professionals. Drugs.com allows users to summarize their overall drug experience via anonymous text narratives and a numerical rating system, with 1 indicating not effective and 10 indicating most effective [30]. The total number of online reviews for a given drug is usually greater than the number of numerical ratings for that drug because not all reviewers chose to contribute a numerical summary rating.

Text narratives and numerical ratings from patient reviews were imported into Microsoft Excel (Microsoft Corporation, Redmond, Washington) for evaluation and analysis. A primary coder read each review to identify language used by the patient to convey an adverse event. Those keywords and phrases were then used to manually select low-level terms (LLTs) within MedDRA (Medical Dictionary for Regulatory Activities), version 18.0 [31]. MedDRA terminology is the international medical terminology developed under the auspices of the International Council for Harmonisation of Technical Requirements for Pharmaceuticals for Human Use (ICH). The corresponding preferred terms (PTs) were dictated by the LLT choice, consistent with MedDRA “Points to Consider” guidelines. Each review was also coded for mention of complaints of drug cost or insurance coverage. Microsoft Excel was then used to randomly select a subset of 166 cases that included at least 10% of the reviews for each of the five drugs. This subset was recoded by two secondary coders working independently and blinded to the LLT and PT selections of the primary coder. The percentage of reviews coded identically between the primary and the secondary coders was 74.7% and 78.3%, respectively. All three coders then met in person to discuss several recurring themes of disagreement apparent in the subset. For example, some reviews included the term “tolerance” to describe a drug effect that was diminished or lost with continued use (“I developed tolerance” or “I gained tolerance”). This was variably coded with the LLT *drug effect decreased* or *drug tolerance increased*. The coders concluded that a clinician best assesses tolerance because it is an advanced concept that may include different pharmacokinetic, pharmacodynamic, and behavioral dimensions. In contrast, the patient is often best positioned to determine whether a hypnotic is still working or if the effect has waned. Therefore, narratives that mentioned tolerance were recoded as the LLT *drug effect decreased*. Reviews mentioning “depression” or “feeling depressed” were similarly problematic. The coders decided that the LLT *depression* refers to a formal, clinical diagnosis describing a set of symptoms persisting for a minimal length of time and that the mood changes implied in such narratives were more appropriately captured with the LLT *depressed mood*. Adjustments based on these and other term selections improved primary-secondary coder PT agreement to 79.6% and 87.3% of cases, within the subset. Those coding adjustments were then implemented across the entire data set. A small number of reviews mentioned or implied recent or concomitant use of other drugs, but we did not attempt to adjust for that in our data because those instances were rare and difficult to interpret. The primary and secondary coders completed MedDRA training workshops and webinars.

### Food and Drug Administration Adverse Event Reporting System

For FAERS data, 11 quarterly reports (2015-Q1 through 2017-Q3) were downloaded from the FDA website [32]. Microsoft Access (Microsoft Corporation) was used to select 49,389 reports for eszopiclone, ramelteon, suvorexant, zaleplon, or zolpidem based on the “product_ai” field of the DRUG file. To limit the size and complexity of the data set, all reports with multiple drugs were excluded by eliminating cases with more than one “primaryid” entry; subsequently, only reports designating one of the five hypnotics as the primary suspect in the “role_cod” field were selected. These were matched back to the REAC, DEMO, and INDI files to retrieve the adverse event PTs, indications for therapy and demographics. For cases that appeared in multiple quarters due to report updating, we utilized the most recent version. This produced 5916 unique reports concerning either eszopiclone (n=196), ramelteon (n=103), suvorexant (n=4095), zaleplon (n=37), and zolpidem (n=1485) as the only drug reported. These were tabulated in Microsoft Excel for further analysis.

### Statistical Analysis

Statistical analyses were performed using R statistical software (version 3.5.1; R Core Team, Vienna, Austria) using a two-sided significance level of .05. Statistical comparison of user drug ratings was performed using the nonparametric Kruskal-Wallis test, followed by the Dunn posthoc for all pairwise comparisons. To determine the relationship between user rating and complaints about efficacy or cost in the narrative, univariate logistic regression models were fit for each of the drugs separately. Logistic regression coefficients were reported as odds ratios with 95% CIs. The count of distinct adverse events recorded from the reviews (not including the PTs *drug ineffective* and *drug effect incomplete*) was analyzed using Poisson regression, with user rating as the explanatory variable. The exponential of the Poisson regression model coefficients was reported as the incidence rate estimates, and robust standard errors were calculated using the Delta method to control for mild violation of the distribution assumption that the variance equals the mean.

### Ethics

The Institutional Review Board at Midwestern University - Downers Grove declared that this project does not qualify as human subjects research. The annotated datasets generated and analyzed during this study are available from the corresponding author on reasonable request.

## Results

### Adverse Event Reporting in Online Reviews Versus Food and Drug Administration Adverse Event Reporting System

The 10 most common MedDRA PT adverse events coded from online patient reviews are shown in Tables 1 and 2, expressed as a percentage of the total number of PTs recorded from all the reviews for each hypnotic. *Drug ineffective* was commonly reported for all five drugs (eszopiclone: 45/319, 14.1%; ramelteon: 24/104, 23.1%; suvorexant: 159/567, 28.0%; zaleplon: 22/82, 27%; and zolpidem: 33/958, 3.4%). Furthermore, partial or limited efficacy was noted in complaints that were coded as *drug effect incomplete* for ramelteon (12/104, 11.5%) and suvorexant (22/567, 3.9%). *Amnesia* was among the top 10 complaints for all three Z-drugs (eszopiclone: 8/319, 2.5%; zaleplon: 4/82, 5%; zolpidem: 161/958, 16.8%). Certain adverse events are notable for each of the different drugs. Zolpidem was frequently associated with complex partial sleep behaviors, including *abnormal sleep-related event* (90/958, 9.4%), *sleep-related eating disorder* (59/958, 6.2%), *somnambulism* (47/958, 4.9%), and *sleep talking* (34/958, 3.6%). Eszopiclone was commonly associated with *dysgeusia* (94/319, 29.5%). Suvorexant reviewers reported distressing parasomnias that included *nightmare* (54/567, 9.5%), *sleep paralysis* (26/567, 4.6%), and *abnormal dreams* (25/567, 4.4%).

Tables 3 and 4 show the top 10 most common PTs in FAERS reports, expressed as the percentage of the total number of PTs for each drug. *Drug ineffective* emerged as the most common PT recorded in FAERS for each of the five drugs (eszopiclone: 78/458, 17.0%; ramelteon: 30/208, 14.4%; suvorexant: 1108/6171, 18.0%; zaleplon: 18/77, 23%; zolpidem: 499/3448, 14.5%). Other PTs common for the individual drugs include *amnesia* (51/3448, 1.5%) and *somnambulism* (75/3448, 2.2%) with zolpidem; *dysgeusia* (22/458, 4.8%) and *product substitution issue* (30/458, 6.6%) with eszopiclone; and *nightmare* (422/6171, 6.8%), *abnormal dreams* (382/6171, 6.2%), and *sleep paralysis* (124/6171, 2.0%) with suvorexant.

**Table 1:** Preferred terms manually coded from online patient reviews of Z-drugs.

#	Eszopiclone (n=319)		Zaleplon (n=82)		Zolpidem (n=958)	
	Preferred term	n (%)	Preferred term	n (%)	Preferred term	n (%)
1	Dysgeusia	94 (29.5)	Drug ineffective	22 (27)	Amnesia	161 (16.8)
2	Drug ineffective	45 (14.1)	Insomnia	5 (6)	Abnormal sleep-related event	90 (9.4)
3	Drug effect decreased	17 (5.3)	Somnolence	5 (6)	Sleep-related eating disorder	59 (6.2)
4	Product substitution issue	17 (5.3)	Amnesia	4 (5)	Drug effect decreased	51 (5.3)
5	Insomnia	10 (3.1)	Abnormal sleep-related event	3 (4)	Somnambulism	47 (4.9)
6	Depressed mood	9 (2.8)	Anxiety	3 (4)	Sleep talking	34 (3.6)
7	Somnolence	9 (2.8)	Hallucination	3 (4)	Drug ineffective	33 (3.4)
8	Amnesia	8 (2.5)	Headache	3 (4)	Drug dependence	32 (3.3)
9	Anxiety	7 (2.2)	Restless legs syndrome	3 (4)	Somnolence	28 (2.9)
10	Headache	6 (1.9)	Abdominal pain upper	2 (2)	Hallucination	27 (2.8)

**Table 2:** Preferred terms manually coded from online patient reviews of ramelteon and suvorexant.

#	Ramelteon (n=104)		Suvorexant (n=567)	
	Preferred term	n (%)	Preferred term	n (%)
1	Drug ineffective	24 (23.1)	Drug ineffective	159 (28.0)
2	Drug effect incomplete	12 (11.5)	Nightmare	54 (9.5)
3	Somnolence	7 (6.7)	Headache	27 (4.8)
4	Dizziness	5 (4.8)	Somnolence	27 (4.8)
5	Insomnia	5 (4.8)	Sleep paralysis	26 (4.6)
6	Abnormal dreams	4 (3.9)	Abnormal dreams	25 (4.4)
7	Depressed mood	4 (3.9)	Drug effect incomplete	22 (3.9)
8	Feeling abnormal	4 (3.9)	Feeling abnormal	20 (3.5)
9	Anxiety	3 (2.9)	Insomnia	19 (3.4)
10	Headache	3 (2.9)	Hangover	17 (3.0)

**Table 3:** Preferred terms retrieved from FAERS reports for Z-drugs.

#	Eszopiclone (n=458)		Zaleplon (n=77)		Zolpidem (n=3448)	
	Preferred term	n (%)	Preferred term	n (%)	Preferred term	n (%)
1	Drug ineffective	78 (17.0)	Drug ineffective	18 (23)	Drug ineffective	499 (14.5)
2	Insomnia	33 (7.2)	Drug effect incomplete	3 (4)	Insomnia	146 (4.2)
3	Product substitution issue	30 (6.6)	Insomnia	3 (4)	Product substitution issue	96 (2.8)
4	Dysgeusia	22 (4.8)	Product quality issue	3 (4)	Somnambulism	75 (2.2)
5	Product quality issue	16 (3.5)	Product substitution issue	3 (4)	Somnolence	59 (1.7)
6	Drug effect decreased	14 (3.1)	Abnormal behavior	2 (3)	Drug dependence	57 (1.7)
7	Delirium	12 (2.6)	Drug hypersensitivity	2 (3)	Amnesia	51 (1.5)
8	Headache	7 (1.5)	Malaise	2 (3)	Road traffic accident	48 (1.4)
9	Nausea	7 (1.5)	Nausea	2 (3)	Toxicity to various agents	47 (1.4)
10	Sleep disorder	7 (1.5)	Nightmare	2 (3)	Overdose	46 (1.3)

**Table 4:** Preferred terms retrieved from FAERS reports for ramelteon and suvorexant.

#	Ramelteon (n=208)		Suvorexant (n=6171)	
	Preferred term	n (%)	Preferred term	n (%)
1	Drug ineffective	30 (14.4)	Drug ineffective	1108 (18.0)
2	No adverse event	17 (8.2)	Nightmare	422 (6.8)
3	Intentional overdose	14 (6.7)	Abnormal dreams	382 (6.2)
4	Toxicity to various agents	13 (6.3)	Somnolence	256 (4.2)
5	Somnolence	8 (3.9)	Headache	199 (3.2)
6	Suicide attempt	8 (3.9)	Feeling abnormal	189 (3.1)
7	Drug prescribing error	7 (3.4)	Hallucination	174 (2.8)
8	Dizziness	6 (2.9)	Insomnia	136 (2.2)
9	Middle insomnia	6 (2.9)	Sleep paralysis	124 (2.0)
10	Drug administered to patient of inappropriate age	5 (2.4)	Adverse event	109 (1.8)

**Figure 1:**
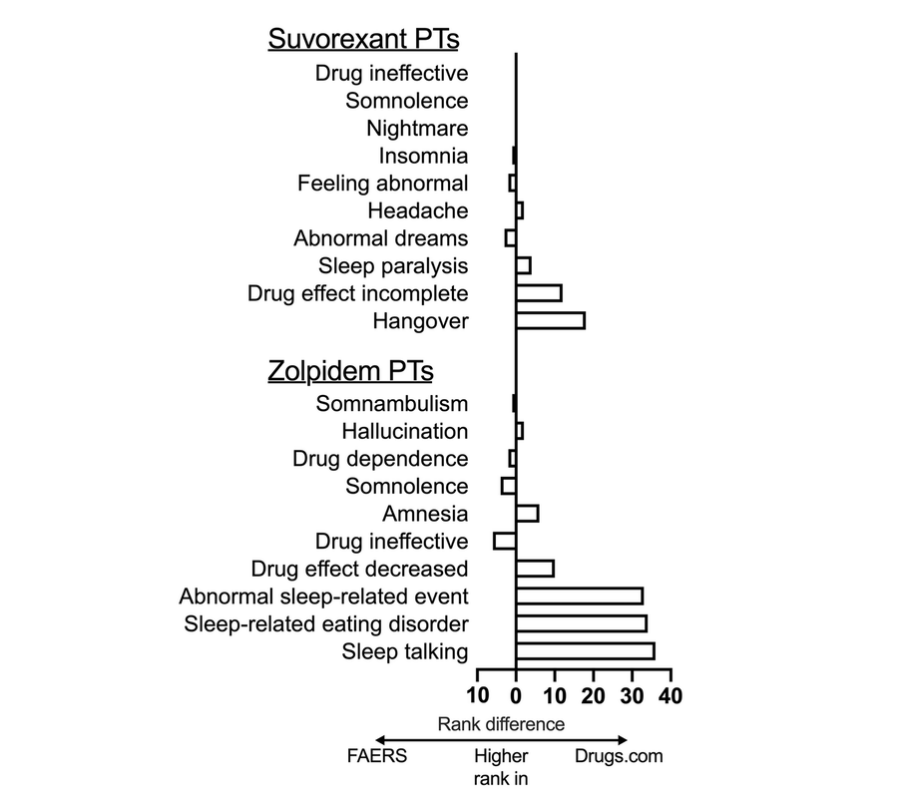
Difference in rank position for the 10 most common PTs in Drugs.com vs their rank in FAERS, for suvorexant and zolpidem. The rank of the Drugs.com PT was subtracted from its corresponding rank in FAERS. Bars extend to the right for PTs that held higher rank position in Drugs.com than in FAERS. For PTs that held higher rank position in FAERS compared to Drugs.com, bars extend to the left. PT: preferred term; FAERS: Food and Drug Administration Adverse Event Reporting System.

To graphically summarize areas of agreement and disagreement between Drugs.com and FAERS, we plotted the difference in rank positions of the most frequent PTs in Drugs.com from their position in FAERS for suvorexant and zolpidem (Figure 1). In this representation, a small bar indicates PTs that were ranked similarly in Drugs.com and FAERS, while a larger bar shows differing ranks between the two lists. For zolpidem, for example, *sleep talking* ranked as the 6th most common PT in Drugs.com, but only the 42nd most common in FAERS; thus, the rank difference is 36. There were insufficient data from one or both sources to provide similar representations for the other three drugs studied.

### Online Review User Ratings

**Figure 2:**
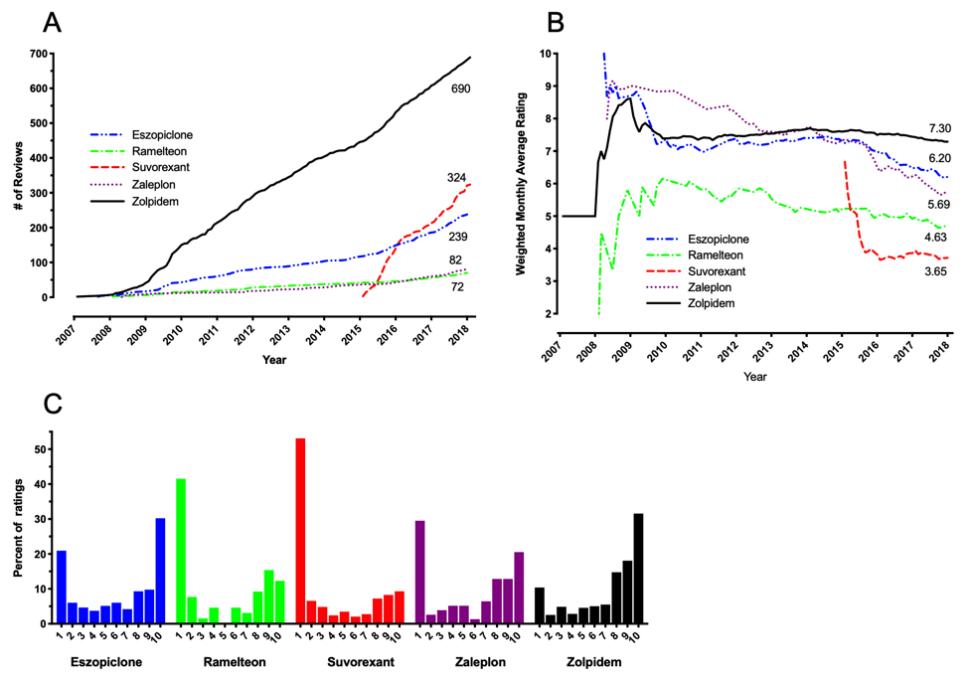
Statistics for consumer reviews of hypnotics on the website Drugs.com. (A) Accumulation of consumer reviews per month. Running total number of reviews are depicted for eszopiclone, ramelteon, suvorexant, zaleplon, and zolpidem. (B) Running monthly average, weighted by frequency, based on reviewers’ numerical summary ranking (1-10), with 1 indicating a very poor experience and 10 indicating a very positive experience. (C) Percent frequency histograms for numerical (1-10) user ratings of insomnia drugs from drugs.com. Data are inclusive of reviews posted between February 2007 and March 2018.

We next considered the Drugs.com online reviews in more detail. Figure 2A depicts the accumulation of reviews for each drug on a monthly basis for the period of February 2007 through March 2018. As of March 2018, zolpidem had the most reviews (n=690), followed by suvorexant (n=324), eszopiclone (n=239), zaleplon (n=82), and ramelteon (n=72). Figure 2B depicts a running monthly average of the numerical 1-10 user ratings. Zolpidem had an average rating of 7.30, followed by the other two Z-drugs, eszopiclone (6.20) and zaleplon (5.69). The nonbenzodiazepine receptor drugs ramelteon and suvorexant were rated 4.63 and 3.65, respectively. Numerical ratings were tabulated in frequency histograms (Figure 2C), which yielded bimodal distributions for each of the five drugs. These data show that a high percentage of reviewers assigned suvorexant and ramelteon the lowest possible score of “1” (154/290, 53.1% and 27/65, 41.5%, respectively). In comparison, only 10.4% (66/637) of zolpidem reviewers rated it as “1,” while 31.6% (201/637) rated it “10.” Analysis of user ratings using Kruskal-Wallis test followed by the Dunn post hoc test found that zolpidem was rated significantly higher than the other four drugs, while suvorexant was rated significantly lower than all the Z-drugs (Table 5).

**Figure 3:**
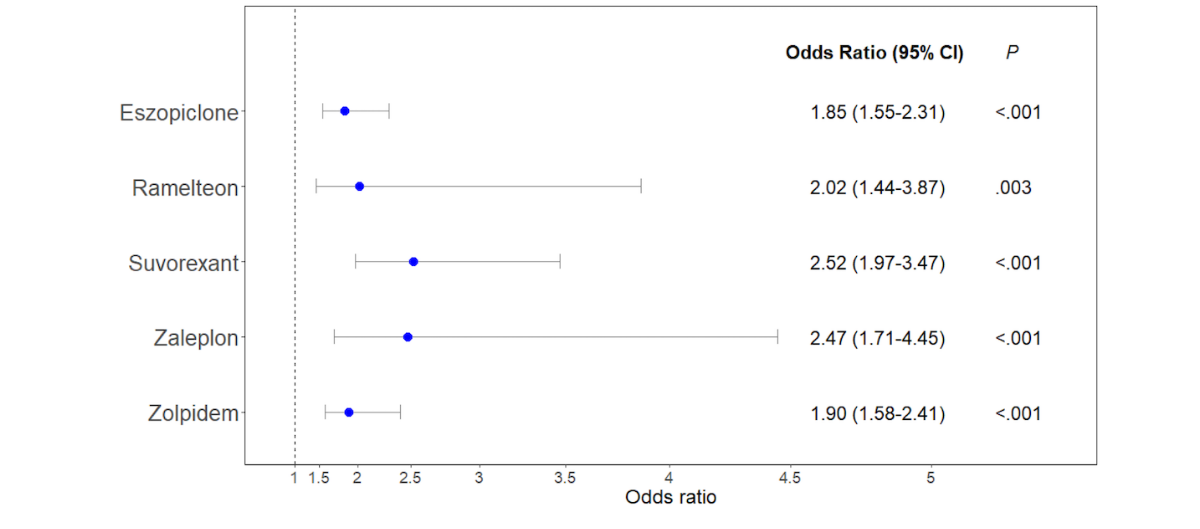
Odds ratio plot of the univariate logistic regression models to assess the relationship between user ratings and patient complaints of poor efficacy, that is, reviews that were coded for *drug ineffective* or *drug effect incomplete*. Odds ratios and 95% CIs are depicted by the circle and whiskers, respectively.

We then explored factors that might contribute to high or low user ratings. Focusing specifically on comments of poor efficacy, we found a statistically significant association between low user ratings and reviews that were coded for the PTs *drug ineffective* or *drug effect incomplete* for all five drugs (Figure 3). The odds ratios represent the increased odds of an ineffective complaint relative to a unit decrease in user rating. The number of reviews that were coded for *drug ineffective* or *drug effect incomplete* was 44/239 (18.4%) for eszopiclone, 25/72 (35%) for ramelteon, 165/324 (50.9%) for suvorexant, 17/82 (20.7%) for zaleplon, and 34/690 (4.9%) for zolpidem. In addition, a Poisson regression analysis found a significant inverse correlation between the numerical rating and the number of distinct PTs captured from a review for all five drugs (Table 6).

**Table 5:** Comparisons of hypnotic user ratings in Drugs.com with Kruskal-Wallis followed by Dunn post hoc.

Drug - comparator (median user rating)	Adjusted *P* value
Ramelteon (3) - eszopiclone (7)	.003
Suvorexant (1) - eszopiclone (7)	<.001
Suvorexant (1) - ramelteon (3)	.10
Zaleplon (7) - eszopiclone (7)	.09
Zaleplon (7) - ramelteon (3)	.11
Zaleplon (7) - suvorexant (1)	<.001
Zolpidem (8) - eszopiclone (7)	.003
Zolpidem (8) - ramelteon (3)	<.001
Zolpidem (8) - suvorexant (1)	<.001
Zolpidem (8) - zaleplon (7)	<.001

**Table 6:** Estimated incidence rate ratios for the effect of user ratings on the number of adverse events coded from patient reviews. The expected count of PTs is multiplied by a factor of the estimated incidence rate ratios when the user rating increases by one unit.

Drug	Incidence rate ratios (95% CI)	*P* value
Eszopiclone	0.89 (0.85-0.92)	<.001
Ramelteon	0.90 (0.83-0.97)	.007
Suvorexant	0.90 (0.86-0.95)	<.001
Zaleplon	0.88 (0.79-0.98)	<.001
Zolpidem	0.88 (0.86-0.90)	<.001

The estimated effect sizes correspond to a percent change in the incidence rate of PTs by 11% in eszopiclone, 10% in ramelteon, 10% in suvorexant, 12% in zaleplon, and 12% in zolpidem for a one-score decrease in the user rating. Considering eszopiclone, for example, this suggests that the expected count of PTs decreased by a factor of 0.89 when the user rating increased by one unit.

Users also mentioned cost in the reviews, most commonly with suvorexant (75/324, 23.1%) and eszopiclone (37/239, 15.5%). Univariate logistic regression showed that the tendency to mention cost did not predict lower numerical ratings for any of the drugs (Multimedia Appendix 1). For eszopiclone, however, each one-unit increase in user rating increased the odds of a comment about cost by 1.24, suggesting that cost commentary may be correlated with a *higher* eszopiclone rating. Zaleplon was not included in this analysis due to insufficient data, with only 1 of 82 reviews mentioning cost.

Drugs.com does not collect demographic data for individual reviewers, but they compile top-level information on user age and country of residence, which was provided to us after sending an email request to their user support (Multimedia Appendix 2). These data show that more than half (54%) of the reviews are contributed by patients aged ≤44 years and that most reviewers are female (57%) and from the United States (70%).

We also extracted from FAERS patient demographics data on indication of therapy and reporter information, which are summarized in Multimedia Appendix 3. A plurality of reports concerned female patients (2935/5916, 49.6%), and the most common indications were sleep disorders (3735/5916, 63.1%). A substantial fraction of the cases did not report gender (1122/5916, 19%), age (4054/5916, 68.5%), or indication (2102/5916, 35.5%). Most cases (4618/5916, 78.1%) were reported by the consumers themselves, while nearly all of the remainder were reported by health care professionals. More than 9/10 FAERS cases were contributed by reporters in the United States (5383/5916, 91%). Patient age was normally distributed around a mean of 56.9 (SD 18.2) years (Multimedia Appendix 4).

## Discussion

### Principal Findings

In general, we found that adverse event data interpreted from patient online reviews were consistent with the adverse event data in FAERS. Both resources show that poor efficacy is common with all five drugs and that amnesia is frequent with Z-drugs. A variety of adverse events particular to the individual drugs were also evident in both, for example, *nightmare* with suvorexant, *dysgeusia* and *product substitution issue* with eszopiclone, and *dizziness* with ramelteon.

The two sources showed good agreement in zolpidem adverse events (*drug ineffective*, *somnambulism*, *somnolence*, *drug dependence*, and *amnesia* were among the top 10 most frequent PTs in both), but the online reviews contained a higher rate of partial sleep activities, such as walking, talking, and eating while semiconscious (for sample reviews, see Multimedia Appendix 5). Reviewers often did not remember those events, which in turn contributed to a high incidence of *amnesia* in the online data versus FAERS data (16.8% vs 1.5%). This discrepancy between the two sources may be explained by the fact that the propensity for zolpidem-induced partial sleep activities is now so well known that health professionals see little value in reporting it, which would lead to an underestimation of those events in recent FAERS quarterlies. The opposite trend may be occurring in the online review data, where many reviewers seemed eager to share those stories. Such perspectives might be expected from a younger population that is more likely to participate in online activity [22,33] and appears to be more heavily represented in Drugs.com compared to FAERS. Indeed, patient age is one of the notable differences between the two resources: The average age in FAERS was nearly 60 years, while over half of the Drugs.com user population is aged ≤44 years. Regardless, the high numerical ratings for zolpidem (Table 5) suggest that online reviewers may be trivializing the very serious dangers of Z-drug impairment [34], which recently prompted an FDA-mandated boxed warning for all three Z-drugs [35]. Other studies have found a general disregard of serious safety issues among the online community. For example, official safety warning announcements regarding zolpidem registered on social media in only limited and transient fashion [36], and online reviewers minimized the dangers associated with sibutramine, a weight-loss drug that was ultimately withdrawn for safety reasons [22]. A related issue was described by Adusumalli et al [24], who noted that patients may tend to rate addictive drugs more highly than alternatives that are less addictive but may be equally effective [24]. This suggests that patient opinion of zolpidem and other Z-drugs might be positively correlated with their habit-forming tendencies.

For suvorexant, we found strong agreement between the two sources. Eight adverse event PTs were among the top 10 most frequent in both sources: *drug ineffective*, *nightmare, somnolence*, *headache*, *sleep paralysis*, *abnormal dreams*, *feeling abnormal*, and *insomnia*. The high incidence of *drug ineffective* is in agreement with the reported incidence in another recent study [37], which found that suvorexant is one of the most common drugs associated with this complaint in the FAERS database. Parasomnias were also frequent, especially nightmares, which deserve special comment because they seem to be rather more intense than a typical “scary” dream, with numerous patients describing them in exceptionally vivid and terrifying themes (Multimedia Appendix 5). Here, it is important to note that nightmare was not mentioned as a potential adverse event in the suvorexant prescribing information [38] nor is it discussed in an exhaustive review of the discovery and clinical development of suvorexant [29]. This discrepancy might be explained if the premarket trials detected only mildly disturbing dreams that were coded not as *nightmare* but as *abnormal dreams—*an adverse event that is explicitly described in the suvorexant prescribing information. However, our findings indicate that conflating *nightmare* with *abnormal dreams* does not provide a true picture of the user experience with this drug. This observation adds to previous studies, which also showed that patient-contributed online data can help capture adverse events that were not described in trial data evaluated by the FDA [39,40].

### Factors Influencing Summary Drug Rating

For all five drugs, negative ratings significantly increased the odds of the PT drug ineffectiveness and the frequency of distinct PTs coded from the text of the corresponding review. A substantial number of online reviews for suvorexant, ramelteon, and eszopiclone explicitly mentioned cost or insurance coverage, a concern that is likely to be more prominent with reviewers in the United States compared to patients from countries with national health care programs. Unexpectedly, we found that commentary about affordability did not predict low user ratings for any of the drugs, and eszopiclone reviewers who commented about cost were actually more likely to assign a *higher* numerical rating. We think this seeming paradox is explained by the advent of generic formulations of eszopiclone in the United States during the review period, which led to complaints of *product substitution issue* from reviewers who perceived less benefit from the generic formulations (Multimedia Appendix 5). Accordingly, that PT did not appear in the eszopiclone reviews until September 2014. Around the same time, the previously stably running average began a gradual downward trend (Figure 3B).

To put the patient ratings in perspective, we considered several unrelated medications noted to cause distressing adverse events. The antipsychotic olanzapine causes significant weight gain and metabolic disorder but has a 6.7 average rating on Drugs.com [41]. Methotrexate, used frequently for rheumatoid arthritis, is associated with gastrointestinal upset, painful mouth ulcers, and fatigue, but has a rating of 7.0 [42]. The antibiotic clindamycin is rated 5.9, despite causing diarrhea in a high percentage of patients [43]. This brief sampling suggests that even drugs with onerous adverse events can earn a positive rating, as long as patients perceive real benefit. The exceedingly low ratings of suvorexant and ramelteon were even more remarkable when viewed in this context. In the larger view, our observations suggest that online patient evaluations of hypnotics are guided by fundamental considerations of drug efficacy and tolerability.

### Patient Perspective and Practical Utility of Online Reviews

A patient-reported outcome is a patient’s assessment of their own response to therapeutic intervention that is not reinterpreted or filtered by a clinician [44,45]. Because patients tend to evaluate their response to therapy in a holistic sense, patient-reported outcomes are useful evaluations of functional status and quality of life. These patient-reported outcomes are collected with validated instruments during drug development trials, but after a drug is marketed, there is a continued need to capture the patient’s opinion in the assessment of therapeutic response. Online reviews seem well suited for recording the patient perspective, especially for drugs like hypnotics, where the balance of efficacy versus adverse events relates directly to the functional benefits that accrue with therapeutic success, that is, improved sleep. Furthermore, hypnotics should be reconsidered with regard to the patient’s perspective because guidance on examining patient-reported outcomes [46,47] was published after trials of several important hypnotic medications were completed [48]. In addition, clinical trial participants differ from real-world patients [49]; therefore, even hypnotics with historical patient-reported outcomes can benefit from continued surveillance of the patient perspective in the form of online reviews.

In the most practical terms, online reviews can serve as an initial source of information for patients seeking a mix of views on the potential advantages and disadvantages of a given drug therapy. Compared to professional resources with similar information, online reviews are written with language and tone that resonate with the average patient [21,50]. The recently introduced FAERS dashboard can also educate consumers about drug adverse events [51]; however, interpretation of those data is still quite challenging for untrained people. Regardless of the resource, health care professionals should be ready to correct a number of common misconceptions. Patients must understand that a report of an adverse event does not establish causality, nor can individual probability of experiencing an adverse event be inferred from adverse event reports. Such a discussion can help the clinician develop an optimal plan of care that fully considers patient concerns about adverse events and expectations of effectiveness.

### Limitations

The most obvious limitation of this study is the suspect validity of patient online data. We observed no reason to doubt the sincerity of these reviews, but the general vulnerability of patient reviews to misinformation and manipulation must be kept in mind, as other investigators have cautioned [52]. The subjective nature of patient self-evaluations may also be an issue, although it should be noted that self-reported data are standard for clinical sleep studies [48], which often rely on the subjects themselves to record their data and observations in a daily “sleep diary” [53].

Inherent differences in size and complexity between the two data sources made it difficult to compare the data pertaining to the same time period. We needed all the online reviews available since Drugs.com introduced this feature (in ~2008); otherwise, those data sets would be too small. In contrast, even just a few years of FAERS data can become overwhelmingly large, and FAERS conventions undergo periodic changes to MedDRA coding practices and formatting of the quarterly files. In view of those complications, we opted for a simple solution that limited our FAERS survey to 11 quarters, following the approval of suvorexant in August 2014. This compromise facilitated a comparison of two sets of concurrent suvorexant data, the agent with the most limited clinical experience. Perhaps, this approach contributed to the high degree of agreement between FAERS and Drugs.com for suvorexant; if so, it probably also explains some of the disagreement for the other drugs, where the data timelines overlap less.

Other limitations concern the sparse nature of the online data compared to reports collected by regulatory agencies [54]. Although Drugs.com collects limited data on their user population, individuals rarely volunteered their age, gender, or medication dosage in the text of their reviews. Furthermore, it is often impossible to know what additional drugs they may have taken concomitantly, which could be the primary source of the adverse event. This consideration is especially relevant to these hypnotics, because all are subject to hepatic metabolism and attendant drug-drug interactions. Because follow-up clarification of case information is not possible with anonymous online testimonials lacking a verifiable patient, these limitations are fixed [55]. In contrast, we noted that many FAERS reports also lacked basic data including age, gender, and indication. Most revealing is the fact that more than three quarters of the FAERS cases (4618/5916, 78.1%) were reported by consumers, which means that these FAERS reports apparently share the same untrained origins as the online reviews, regardless of subsequent coding and processing by professionals.

### Conclusions

Our work adds to a growing body of literature that has explored the utility of online reviews. Patient numerical ratings of hypnotic medications were influenced by perceptions of efficacy and adverse events, but not by cost. Patients rated zolpidem the highest, while ramelteon and suvorexant were held in relatively poor regard. Patient online reviews emphasized many of the same hypnotic adverse events that are reported to the FDA, most notably lack of efficacy. Future research is needed to determine how online reviews may help collect patient-reported outcomes in the postmarketing realm. Because our results show that online reviews are guided primarily by considerations of drug efficacy and tolerability and match well with adverse event data reported to FAERS, we conclude that patient online reviews offer a valuable supplement to traditional adverse event reporting systems.

## Appendix

Multimedia Appendix 1 Estimated odd ratios for the relationships between cost complaints and user ratings.

Multimedia Appendix 2 Top-level user demographics for drugs.com, as provided by drugs.com user support.

Multimedia Appendix 3 Demographics of 5916 patient reports for five insomnia drugs in the Food and Drug Administration Adverse Event Reporting System. Unkn/NA: Unknown or not available; Sleep dis: Sleep disorders.

Multimedia Appendix 4 Histogram of patient age data in Food and Drug Administration Adverse Event Reporting System reports (mean 56.9 years, SD 18.2 years; range 0-97 years). Age data were available for 31.5% (1862/5916) of reports. Cases recorded in nonyear units were converted to years. For cases recorded in decade units, the midpoint year was used, eg, “7 DEC” was considered 65 years of age.

Multimedia Appendix 5 Example reviews from Drugs.com for eszopiclone, suvorexant, and zolpidem.

## Abbreviations

FAERS: Food and Drug Administration Adverse Event Reporting System
LLT: low-level term
MedDRA: Medical Dictionary for Regulatory Activities
PT: preferred term
