# Supplementary material for: Adverse Events Due to Insomnia Drugs Reported in a Regulatory Database and Online Patient Reviews: Comparative Study
Source: J Med Internet Res. 2019 Nov 8;21(11):e13371. doi: 10.2196/13371 (PMC6874799; doi:10.2196/13371)
Supplement: Multimedia Appendix 1 [file jmir_v21i11e13371_app1.pdf]

Multimedia Appendix 1. Estimated odd ratios (OR) for the relationships between cost complaints and user ratings.

**Drug**	**Reviews Mentioning Cost**	**OR (95% CI)**	***P*-value**
Eszopiclone	37/239, 15.5%	1.24 (1.10-1.42)	<.001
Ramelteon	8/72, 11%	0.97 (0.78-1.18)	.75
Suvorexant	75/324, 23.1%	0.97 (0.89-1.05)	.51
Zolpidem	19/690, 2.8%	1.02 (0.86-0.90)	.85
