# Supplementary material for: Adverse Events Due to Insomnia Drugs Reported in a Regulatory Database and Online Patient Reviews: Comparative Study
Source: J Med Internet Res. 2019 Nov 8;21(11):e13371. doi: 10.2196/13371 (PMC6874799; doi:10.2196/13371)
Supplement: Multimedia Appendix 2 [file jmir_v21i11e13371_app2.pdf]

Multimedia Appendix 2. Top-level user demographics for Drugs.com, as provided by Drugs.com user support.

Gender	%	Age (yrs)	%	Country	%
Female	57%	18-24	7	U.S.	~70
Male	43%	25-34	26	Other	~30
		35-44	21		
		45-54	18		
		55-64	16		
		65+	12		
