# Supplementary material for: Adverse Events Due to Insomnia Drugs Reported in a Regulatory Database and Online Patient Reviews: Comparative Study
Source: J Med Internet Res. 2019 Nov 8;21(11):e13371. doi: 10.2196/13371 (PMC6874799; doi:10.2196/13371)
Supplement: Multimedia Appendix 3 [file jmir_v21i11e13371_app3.pdf]

Multimedia Appendix 3. Demographics of 5916 patient reports for five insomnia drugs in FAERS.

**Gender**	**n (%)**	**Indication**	**n (%)**	**Reporter**	**n (%)**	**Country**	**n (%)**
Female	2935 (49.6)	Sleep dis.	3735 (63.1)	Consumer	4618 (78.1)	U.S.	5383 (91)
Male	1859 (31.4)	Unkn/NA	2102 (35.5)	Physician	511 (8.6)	Japan	217 (3.7)
Unkn/NA	1122 (19)	Other	79 (1.3)	Other	399 (6.7)	Other/NA	212 (3.6)
				Pharmacist	286 (4.8)	Italy	67 (1.1)
				N/A	98 (1.7)	France	37 (0.6)
