# Supplementary material for: Adverse Events Due to Insomnia Drugs Reported in a Regulatory Database and Online Patient Reviews: Comparative Study
Source: J Med Internet Res. 2019 Nov 8;21(11):e13371. doi: 10.2196/13371 (PMC6874799; doi:10.2196/13371)

Multimedia Appendix 4. Histogram of patient age data in FAERS reports. Mean 56.9 years, SD 18.2; range 0-97. Age data were available for 31.5% (1862 of 5916) of reports. Cases recorded in non-year units were converted to years. For cases recorded in decade (DEC) units, the midpoint year was used; e.g. “7 DEC” was considered 65 years of age.

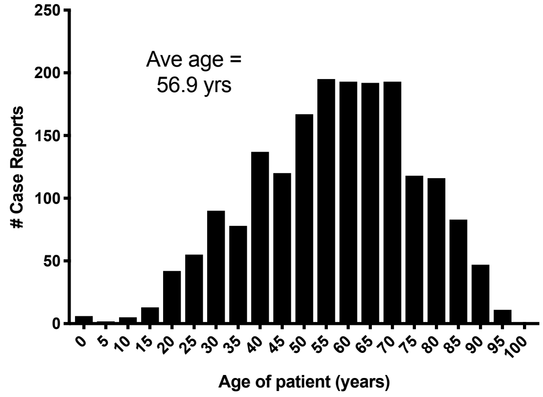

Supplement: Multimedia Appendix 4 [file jmir_v21i11e13371_app4.pdf]
