# Supplementary material for: Adverse Events Due to Insomnia Drugs Reported in a Regulatory Database and Online Patient Reviews: Comparative Study
Source: J Med Internet Res. 2019 Nov 8;21(11):e13371. doi: 10.2196/13371 (PMC6874799; doi:10.2196/13371)
Supplement: Multimedia Appendix 5 [file jmir_v21i11e13371_app5.pdf]

Multimedia Appendix 5. Example reviews from Drugs.com for eszopiclone, suvorexant, and zolpidem.

Eszopiclone-associated product substitution issue

> ESZ 108. “Brand name Lunesta works like a charm. I get 8-10 hours of sleep a night. I have never built up a tolerance and I have never had episodes where I did not remember things if I didn't go to bed right away. I have fibromyalgia and REM sleep is very important. Last month I got the generic brand and I haven't had a good nights sleep since. I can't fall asleep at all. If I start to drift off my brain suddenly wakes up. I'm not even getting 2 hours of sleep a night and that is after laying there till it's almost time to get up and I take 2 muscle relaxers to get a little sleep. Now my pain is unbearable”

> ESZ 111. “Terrible insomnia, took the brand name for years and got sleep, on my second month of the generic and now its not working, At first the generic was working and I was groggier in the morning now a few weeks later I am not getting the knock out effect and waking after less than 5 hours last night I maybe slept 2 hours if at all...”

> ESZ 133. “Okay, the original lunesta was extremely effective, I received samples. For 330 month I couldn't get prescription. So years later they have a generic version. I got a months worth for 111$$. At 3 mg nothing......so I try 6mg.....nothing no bad or good to report! These pills are FRAUD!!!! I'm well accustomed to meds and the generic I took did nothing. I'd get more effect from sugar pill!! I'm getting this so others can see not all generics are equal. There are various companys. I didn't know this, so if you suspect your med isn't up to particular like it used to be.....check the maker!! I'm very upset about this. I'm out $111 for the month for a fake pill made by the company”

> ESZ 173. “I have been taking generic eszopiclone 3mg for several years and haven't had a problem until I the pharmacy changed manufacturers to Orchid. The Orchid pill does not put me to sleep. I have had no problem with Teva manufactured eszopiclone. It works just like it's should. Within 30 mins of taking it, I'm asleep. A year ago I was given Dr Reddys manufactured and it did not put me to sleep either. So far the only generic that works for me is Teva. I have a flag on my account at the pharmacy that I prefer Teva but they apparently ran out of it last week!!? They gave me Orchid and I haven't had a good nights sleep since.It's really crazy to have an approved generic that doesn't work”

> ESZ 181. “Started taking Lunesta when I was 15. The brand name works amazingly! Now that insurance doesn't want to cover the brand without a fight,even if they cover I have

a 100$ Co pay on it!! I'm stuck with the generic and it's not so great. Definitely doesn't work as well plus the orange generic doesn't work at all, I have to have the blue ones to get any type of sleep”

Suvorexant-associated nightmare

SUV 030. “the dreams were axe murderer vivid”

SUV 032. “Its very strange at first, serious nightmares, its almost like lucid dreaming, very 3D, and you have more ability to make decisions in the dream”

SUV 033. “I had 3 hrs of horrible, vivid nightmares that woke me up every 5 minutes panting & sweating”

SUV 037. “This is strange stuff, be careful. You feel tired but it does not shut your mind off, I laid in bed for 4 hours awake. At that point after 2 nights of not sleeping I think I would have fallen asleep any way from exhaustion. I did not think I was asleep but started to experiencing the strangest nightmares I have ever had. After another hour of this I did fall into a dream state sleep for sure. The dreams were extremely unusual and lucid …very strange. I fell in and out of sleep for about 2 hours then woke at 4 am and that was it."

SUV 043. “I have just experienced the worse night of my life due to 10 mg Belsorma. I read reviews, thought they had to be exaggerated..this medicine should not have been approved. Horrific hallucinations/nightmares.. Didn't know if I was awake or asleep . Terrifying. If you are contemplating using this drug, do not do it!!”

SUV 047. “…had the most disturbing dream that kept waking me up and continuing when I fell back asleep. I'm not sure I want to try it again, and I don't recommend.”

SUV 059. “…the weird nightmares. I had hours of vivid nightmarish dreams. Not good. Woke up exhausted. Why would anyone take this medication if it's going to give you nightmares? I'm already an insomniac. I don't need nightmares and hallucinations too….”

SUV 073. “… give me nightmares. Not just standard nightmares- like the worst possible nightmares, vivid and frightening nightmares for hours straight. Then I would wake multiple times and be wide awake….”

Zolpidem-associated amnesia and abnormal sleep-related event

ZOL 035. “I have been taking Ambien for over 2 years now and other than doing things and not remembering about doing it later I love it. But it's not for everyone. My hubby can take it and be out for 2 days straight so be careful.”
ZOL 044. “My experience with this drug has been totally different from most of the posters. I had weird dreams, woke up after 2-3 hours, and with a higher dose- I went into the kitchen and ate a box of donuts and didn't remember any of it the next day.”

ZOL 045. “I think I have the story of all stories about Ambien. I used to take it, and I loved it. I woke up refreshed without any strange things happening. Until one night I had taken a few. I took one and I had built up a tolerance for it so I took another. Next thing I know, I'm naked out in my backyard and I couldn't remember why. Then I come in the house, and there were cops there. I got upset because A) I was naked and B) I didn't know why they were there. Apparently I went nuts, and started throwing things at my boyfriend, and he didn't know what was wrong with me so he called the police. Then they were cuffing me and I was struggling because I could not remember our fight what so ever.”

ZOL 049. “I have been taking Ambien for about 2 years and I love it. I was in the Navy and now I work a 12 hour night shift so I usually can't sleep or if I fall asleep I can't stay asleep long enough. I have had a few nights I do things and don't remember but overall it is wonderful.”

ZOL 063. “I love zolpidem, it works great for me. I've been using it about 4 times a week for 9 months and so far I haven't had any tolerance or loss of effectiveness. I'm asleep within 30 minutes after taking it, every time. I have noticed that it makes me drowsy for a while the next day (not as bad as Tylenol PM or Unisom can). There has also been a couple times when I have woken up in the middle of the night and done weird stuff that I don't remember, which is kind of scary. Overall though it has been a God send.”

ZOL066. “I have been taking zolpidem for 5 months now. At first I started out with making the phone calls, or sending emails and not remembering. Also, I would have a full pack of cigarettes, wake up the next morning and only have a few left. Those effects lasted only the first month of taking it. I still occasionally go through the cigarettes without knowing it but if you want a good, solid night of sleep, I would highly recommend it.”
